# Advances on marine-derived natural radioprotection compounds: historic development and future perspective

**DOI:** 10.1007/s42995-021-00095-x

**Published:** 2021-05-12

**Authors:** Reinu E. Abraham, Mousa Alghazwi, Qi Liang, Wei Zhang

**Affiliations:** 1 Centre for Marine Bioproducts Development, College of Medicine and Public Health, Flinders University, Bedford Park, SA 5042 Australia; 2 Department of Pharmaceutical Sciences, College of Clinical Pharmacy, King Faisal University, Al-Ahsa, Kingdom of Saudi Arabia; 3 Shanxi University of Traditional Chinese Medicine, Taiyuan, 030619 China

**Keywords:** Radio-protective, Therapeutics, Natural compounds, Macro/microalgae, Marine organisms, Mechanism

## Abstract

Natural extracts and compounds from marine resources have gained intensive scientific and industry attention for radioprotective activities in the past ten years. However, the marine-derived radioprotectants have been studied against UV-rays, gamma (γ)-rays and X-rays for more than 30 years. This review aims to identify key marine-derived extracts/compounds and their modes of action studied for radioprotective activities from 1986 to 2019. A comprehensive survey was conducted to establish the trend in terms of the publications each year and the countries of origin. A total of 40 extracts and 34 natural compounds showing radioprotective activities against UV-rays, gamma (γ)-rays and X-rays were identified from a range of marine plants and animals. These extracts and compounds are broadly categorized into polysaccharides, phlorotannins, carotenoids and mycosporine-like amino acids (MAAs). Macroalgae and microalgae were found to be the dominant sources of polysaccharides, phlorotannins and carotenoids. MAAs were mainly identified in algae, sponges, sea cucumber and corals that showed significant UV-absorbing activities. A number of radioprotective mechanisms were shown by these compounds, predominantly free radicals scavenging, inhibition of apoptosis, UV-ray absorption and DNA damage-repair signaling pathways. While these bio-discoveries warrant further investigation and development of radioprotective therapeutics, however, the lack of clinical studies is a major obstacle to be tackled in the future.

**Supplementary Information:**

The online version contains supplementary material available at 10.1007/s42995-021-00095-x.

## Introduction

Over the past few decades, the radiation levels in the atmosphere have rapidly increased due to pollution, penetration of space rays, climatic change, exposure of radioactive chemicals, industries, nuclear power plant and electronic devices. Radiation causes life-threatening side effects to human body and primarily known for causing cancer. The continuous exposure of human body to harmful radioactive rays leads to the formation of reactive oxygen species (ROS) (Xu et al. 2018) that penetrate into the cells and react with DNA, membranes, and proteins, resulting in their dysfunction and cell death (Letsiou et al. 2017; Park et al. 2008). Radiation therapies and radioprotectants are commonly used to treat cancer and safeguard healthy tissues, respectively, from the toxicity of radiation (Abshire and Lang 2018; Connell and Hellman 2009). Radioprotectants are chemical compounds that are designed to protect normal cells from the effect of carcinogenic and toxic substances created by radiation. They can be sourced from synthetic chemicals (Kuntić et al. 2013), and natural products from terrestrial plants and marine organisms (Lupetti et al. 2003).

Marine-derived natural extracts/compounds are widely acknowledged for their structure diversity, novelty and bioactive properties. Phytochemicals including polysaccharides, pigments, phlorotannins, proteins and peptides, and mycosporine-like amino acids (MAA) have been reported to show radioprotective activities (Chrapusta et al. 2017; Oh et al. 2016; Pangestuti et al. 2018). Though many studies have investigated the radioprotective capacity of marine-derived extracts/compounds but their application in pre-clinical and clinical trials are still limited (Oh et al. 2016; Thomas and Kim 2011). A huge scientific gap exists in identifying promising marine-derived extracts and compounds and their unique modes of action that could feed into next level of research or potentially clinical trials (Blunt et al. 2013).

This review aims to provide an in-depth insight into the historical development of marine-derived radioprotective extracts/compounds reported in more than 30 years since 1986, and the trends and future development perspective.

## Historical trend of marine-derived radioprotective compounds

**Fig. 1:**
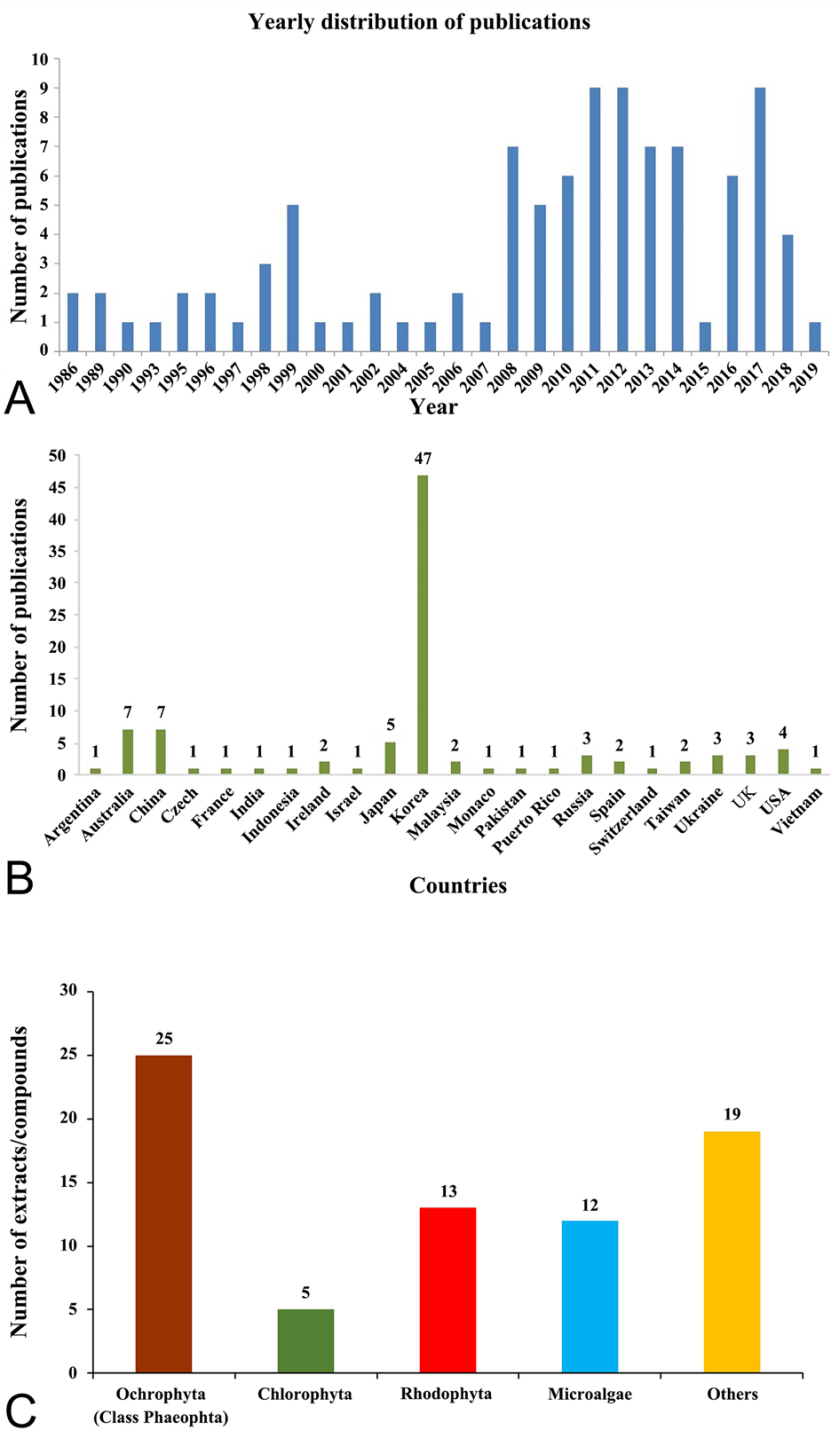
The trend of publications reported from 1986 to 2019 on radioprotective extracts and compounds from marine resources: **a** number of publications reported each year from 1986 to 2019; and **b** number of publications reported by researchers from different countries; and **c** distribution of extracts and compounds from different marine organisms. Footnote: a general survey was conducted through major search engines including Google Scholar, Science Direct, PubMed, Web of Science and Scopus

**Fig. 2:**
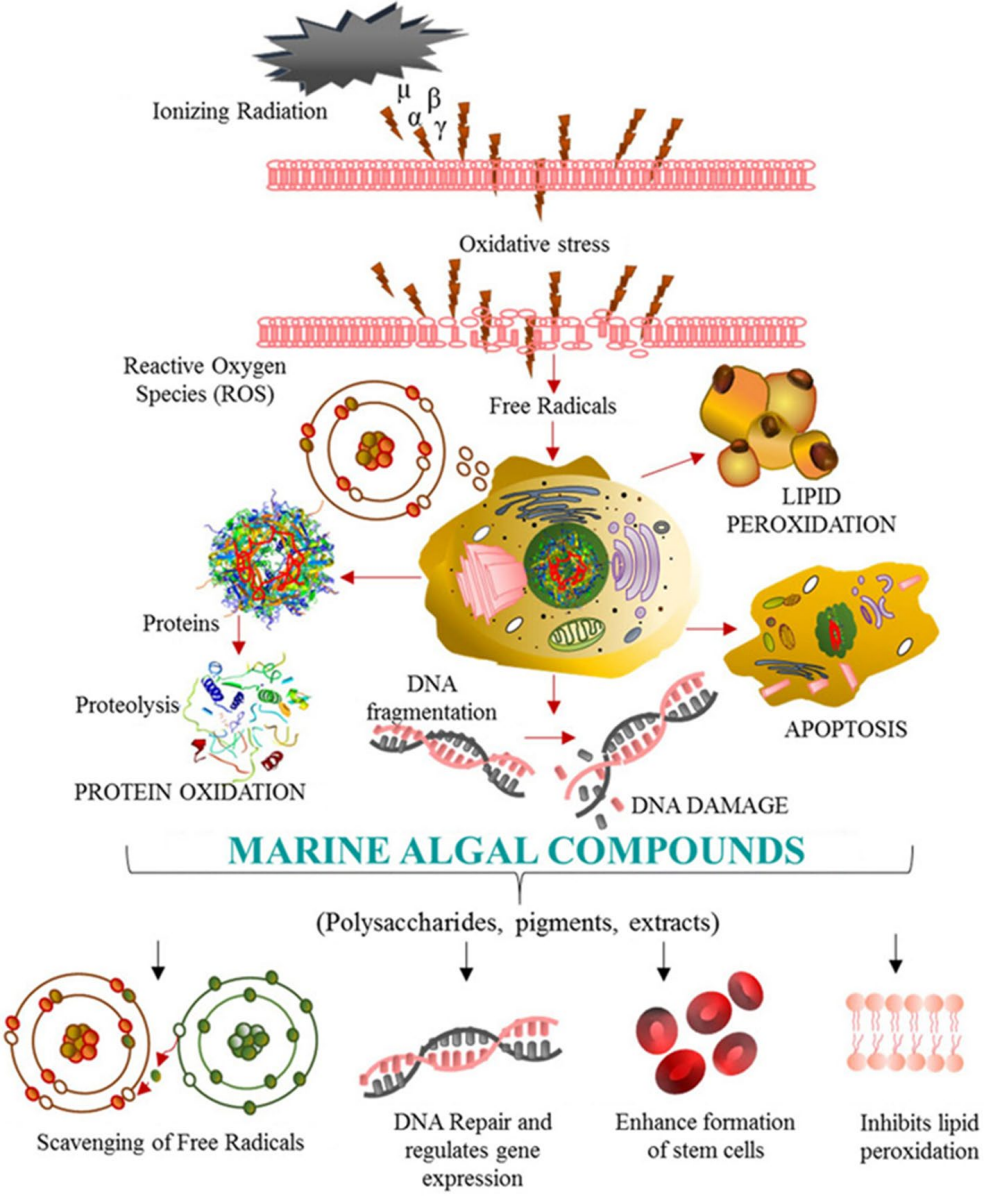
Distribution of studies on radioprotective mechanisms of action of marine-derived extracts and compounds against cell damage due to ionizing radiation

The trend of publications on radioprotective extracts and compounds from marine resources on yearly basis is summarized in Fig. 1a, and the country of origin for the research being conducted (Fig. 1b) from 1986 to 2019. The review highlights the primary sources of key marine radioprotectants being isolated from macro/microalgae, marine microbes, sponges, corals and sea cucumbers. It further shows that countries such as Korea, Australia, China, Japan, and USA have carried 71% of studies in this field when compared to the rest of the world (29%). A total of 40 extracts and 34 compounds (Fig. 1c) were identified from different marine organisms that have shown radioprotective activity in the last 34 years. Pre-clinical studies of these extracts/compounds were predominantly done on gamma (γ)-ray and UV-ray-induced cell damage that leads to oxidative stress or cell death. The mechanisms of these radioprotectants presume to originate by suppressing ROS levels and later follow complex pathways to repair dysfunction of cells caused by DNA, protein and lipid oxidation damage as shown in Fig. 2 (Wu et al. 2020). These marine compounds/extracts were reported to not only suppress the signaling pathways but play a vital role in the development of immune modulatory responses which is essential for therapeutic uses.

## In vitro and in vivo studies based on radiation-induced damage

The electromagnetic waves or particles from radioactive isotopes are known as ionizing radiation (IR) which can be classified as α-, β- and γ-rays. Alpha (α)-rays are emitted as alpha particles from radioisotopes, beta (β)-rays are emitted with high energy electron from radioactive nuclei, and gamma (γ)-rays are electromagnetic waves (Painuli and Kumar 2016). X-rays and γ-rays are photon energy packets that are emitted from the nucleus of radioactive atom when high speed electron changes its direction (Kamran et al. 2016; Lakhwani et al. 2019). Despite the fact that the radiation therapy is widely used to treat cancer, its side effects are well known. For this reason, the radiotherapeutic treatment and radiation dosage (low-to-moderate) should be carefully designed for the patient as it leads to the potential risk of circulatory diseases (Little et al. 2012).

Marine organisms possess natural chemical compounds which show protection against harmful radiations and multiple radioactive contaminations from industries and nuclear plant (Batlle 2012). A number of in vitro and in vivo studies were carried on marine-derived compounds to understand their radioprotective activity and mechanism against different ionizing radiations (Chrapusta et al. 2017). These compounds are primarily extracted from macroalgae, microalgae, sponges, sea cucumber, corals, marine microbes and other marine animals. Based on major radiation types, detailed summary and discussion on the discovery and potential applications are presented below to understand the advancements and trend.

**Fig. 3:**
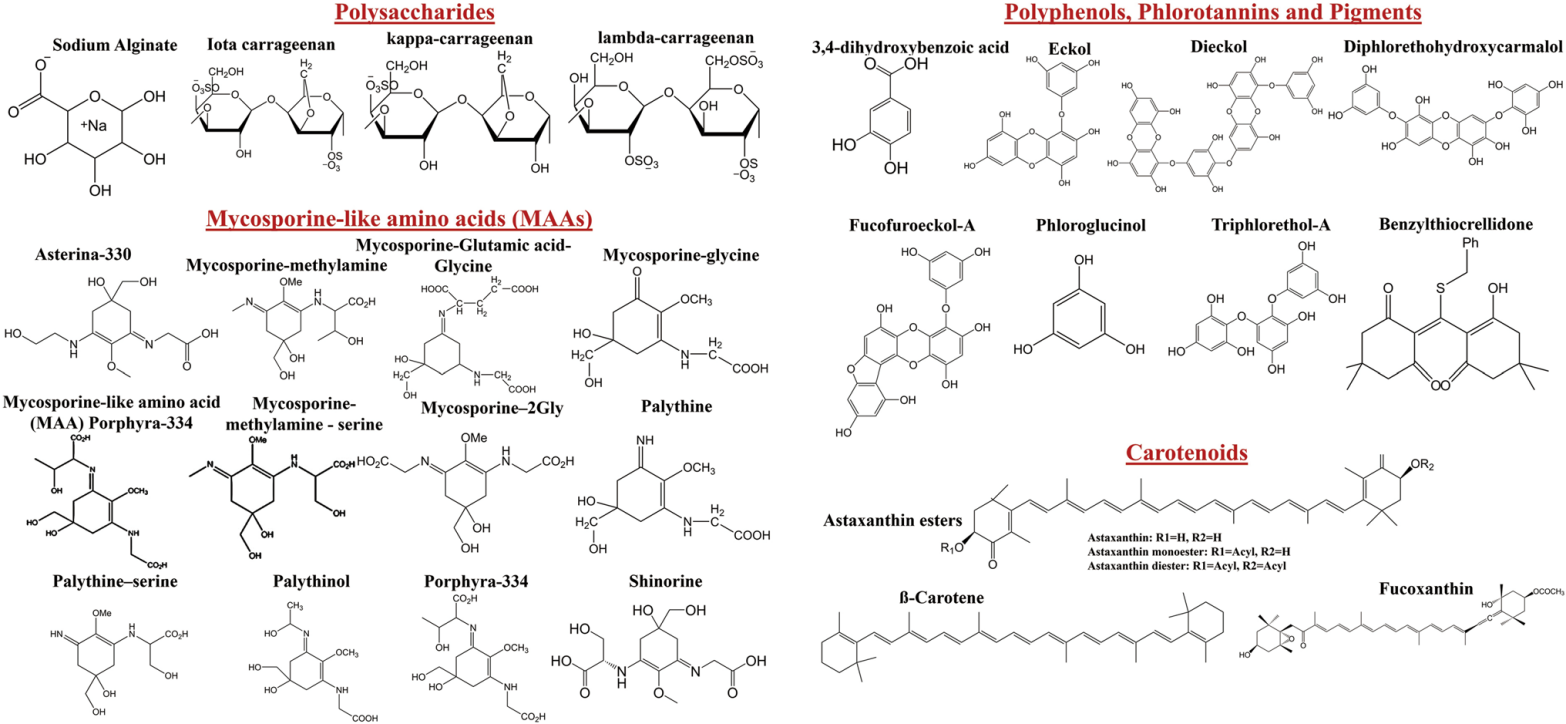
Chemical structures of key radioprotective compounds derived from marine organisms

The chemical structures of some dominant and extensively studied radioprotective compounds are shown in Fig. 3. These polysaccharides block the formation of ROS and play a crucial role in protecting the cells from scavenging activities. Besides polysaccharides there are other marine phytochemicals (e.g., phlorotannins, carotenoids, MAAs) that show potential radioprotective activities using antioxidant properties, and ROS-scavenging activity to protect cells from radiation-induced toxicity (Fig. 2). Phlorotannins such as eckol, phloroglucinol, and dieckol are polyphenolic compounds that are mainly present in macroalgae (brown algae) and highly hydrophilic due to the presence of OH group (Fig. 3) (Fernando et al. 2016). The chemical investigation of marine organisms such as microalgae, macroalgae, microbes, sponges, sea cucumber and corals has shown the presence of radioprotective compounds such as carotenoids (β-carotene, fucoxanthin, zeaxanthin and astaxanthin), mycosporine-like amino acids (MAAs). Synthesis of ultraviolet (UV)-absorbing compounds such as MAAs is one of the photoprotective mechanisms of marine organisms that eliminates the effect of ROS and their oxidative stress (Llewellyn and Airs 2010; Rosic and Dove 2011).

### Gamma (γ) radiation

Gamma (γ) radiation was extensively used in both in vitro and in vivo studies to identify extracts/compounds having radioprotective activity and to further study their mechanistic pathways to repair the cell damage. Patients receiving gamma radiation as part of their radiotherapy are at higher risk such as root dental caries (Campos Velo et al. 2017). Breast cancer imaging such as breast-specific gamma imaging (BSGI) was also reported to increase the risk of breast cancer (Hendrick 2010). It is clear from the data summarized in Table 1 that macroalgae is the most common source of either marine extracts or compounds with radioprotective activities against γ radiation. The only other source is microalgae but exhibiting this activity by different mode of action. A total of eight extracts (Table 1) and seven compounds (Suplementary Table S2) of radioprotective activities were isolated from macroalgae, while microalgae species contributed four extracts and two compounds.

**Table 1:** Summary of extracts derived from marine organisms showing radioprotective activities against γ radiations

Extract	Source	Effective dosage	Radio-protective activity/mechanism	References
Polysaccharide extract	Brown algae: *Ecklonia cava* (1)		Reduced intestinal inflammation of mice model after exposing to γ radiation via inhibiting and reducing iNOS level and paneth cells	Oh et al. (2013)
	Brown algae: *Lactobacillus brevis-*fermented *Ecklonia cava* (2)	150 µg/ml	Protected primary murine splenocytes from γ radiation Reduced DNA damage and ROS production Modulated the expression of Bax, p53 and Bcl-2	Lee et al. (2013b)
	Brown algae: *Laminaria japonica* (3)	100 mg/kg	Recovered reproductive system damage and mating dysfunction induced by γ radiation	Qiong et al. (2011)
	Microalgae: *Spirulina platensis* (4)	12 mg/kg	Increased the level of hemoglobin, white blood cells and red blood cells induced by γ radiation in dogs	Zhang et al. (2001)
Ethanol extract	Microalgae: *Spirulina platensis* (5)	1–5 mg/kg	Provide radioprotective to rats exposed to γ radiation	Mazo et al. (1999)
Ethyl acetate extract	Red algae: *Callophyllis japonica *(6)	100 mg/kg	Improved survival rate of BALB/c mice exposed to γ radiation (12 Gy) via inhibiting lipid peroxidation	Shin et al. (2010)
Methanol extract	Red algae: *Polyopes lancifolia* (7)	100 mg/kg	Increased the survival rate and inhibited the apoptosis induced by γ radiation in jejunal crypt cells of BALB/c mice	Jeong et al. (2011)
Water extract	Brown algae: *Hizikia fusiforme* (8)	6.3 µg/ml	Protected splenocytes of C57BL/6 mice from γ radiation (1.5 Gy) via inhibiting apoptosis and DNA damage	Kim et al. (2015)
Aqueous extracts (Ivastimul)	Microalgae (9)		Provided radio resistance activity against γ radiation by increasing mice survival rate	Vacek et al. (1990)
Aqueous extract (Ivastimul)	Microalgae: *Chlorella* (10)		Increased the survival rate of mice that were exposed to γ radiation	Rotkovska et al. (1989)
Hexane extract	Red algae: *Callophyllis japonica *(hexane and ethyl acetate extracts) (11)	100 mg/kg	Increased survival rate of mice exposed to γ radiation	Kim et al. (2008)
Other extract	Brown algae: *Lactobacillus plantarum-*fermented *Ishige okamurae *(AP2 fraction: high glucose and mannose content) (12)	6.25 µg/ml	Increased survival rate of zebrafish model and reduced malformations against γ radiation (20 Gy) Reduced ROS and NO in the gamma ray-irradiated zebrafish	Lee et al. (2017)

Macroalgae have different radioprotective activities. Brown algal extract of *Hizikia fusiforme* at 6.3 µg/ml inhibited apoptosis and DNA damage in C57BL/6 mice and showed its ability to protect splenocytes when exposed to 1.5 Gy of γ radiation (Kim et al. 2015). Extracts from red algae such as *Callophyllis* were also reported to be radio-protective. Ethyl acetate fraction from *Callophyllis japonica* increased the survival rate of mice exposed to γ radiation (Kim et al. 2008) and inhibited lipid peroxidation in BALB/c mice when exposed to γ radiation (12 Gy) (Shin et al. 2010).

Microalgae extracts showed similar radioprotective activities through multiple mechanisms. An earlier study used ethanol extract (1–5 mg/g—fed 3 times, 4–5 h interval) of *Spirulina platensis* on the bone marrow cells of mouse against γ-rays (250 rad, dose-rate of 48 rad/min). This study showed the reduction of micronucleated bone marrow cells due to its antimutagenic capacity and repaired stimulations (increased polychromatic erythrocyte ratio) (Mazo et al. 1999). *Chlamydomonas reinhardtii* has also demonstrated its radioprotection against 6-h-long γ-radiation (0.49–1677 mGy/h) exposure by reducing ROS formation under oxidative stress, activating oxidative defence system, alterations in mitochondrial metabolism and photosystem II (PSII) efficiency in energy transfer (Gomes et al. 2017).

#### Carotenoids

There are only two carotenoids reported with radioprotective activities. Astaxanthin was reported to have radioprotective activities in four studies. These four studies were conducted on astaxanthin with active concentration of 20 µg/ml using human blood lymphocytes and 50 mg/kg using C57BL/6 mice. The first study investigated the effect of astaxanthin on human peripheral blood lymphocyte (PBL) from people who were exposed to Chornobyl accident. Astaxanthin were treated on PBL at 20 µg/ml astaxanthin and then PBL were γ-irradiated at 1 Gy. The results showed that astaxanthin has radioprotective activity by reducing unstable cytogenetics markers (Kurinnyi et al. 2016). The second study used human blood lymphocytes exposed to 1 Gy of γ radiation, and found that astaxanthin at 20 µg/ml has the ability to reduce DNA damage and apoptosis level (Kurinnyia et al. 2017). The third study was conducted on human peripheral blood lymphocytes. It showed that astaxanthin at 20 µg/ml can reduce the mutagenic effect caused by γ radiation (Pilinska et al. 2016). The last study that was conducted using C57BL/6 mice and showed that astaxanthin can protect hematopoietic system against 8 Gy of γ-ray that induces bone marrow suppression via reducing apoptosis and ROS levels (Xue et al. 2017). The second carotenoid that demonstrated radioprotective activities against γ-ray is β-carotene. A human study was conducted on children who were exposed to radiation from Chernobyl accident. The results demonstrated that giving subjects a diet with 40 mg of β-carotene could reduce the level of lipid peroxidation after a period of 1–3 months (Ben-Amotz et al. 1998). Based on the drug likeness using Lipinski rule of fives (molecular weight ≤ 500 g/mol, log*P* ≤ 5, hydrogen bond donor ≤ 5, and hydrogen bond acceptor ≤ 10) on these phlorotannin compounds (Lipinski et al. 1997), both astaxanthin and β-carotene have larger molecular weights and high LogP which make them unlikely to become drug candidates.

#### Phlorotannins

A total of ten studies reported that six different phlorotannin compounds (eckol, diceckol, diphlorethohydroxycarmalol, fucofuroeckol A, phloroglucinol, and triphlorethol A) as radioprotectant by inhibiting ROS, Bax and caspase, and repairing DNA damage (using γ radiation). Eckol is a phlorotannin that is largely present in brown algae (Fernando et al. 2016), with potential radioprotective activity reported in three studies. Studies show that eckol can provide protection against γ radiation in C57BL/6 mice by inhibiting P53 and Bax, and increasing Bcl-2 expression (Park et al. 2008), protecting V79-4 cells from γ radiation (10 Gy) induced cytotoxicity by inhibiting ROS and apoptosis (Zhang et al. 2008), and inhibiting ROS in ICR mice exposed to γ radiation (Moon et al. 2008). Phloroglucinol is a phlorotannin extracted from brown algae that showed radioprotective activity in five studies. It was showed to protect ICR mice (Moon et al. 2008); C57BL/6 mice (Park et al. 2011); small intestinal crypt cells in C57BL/6 mice (Ha et al. 2013); hamster lung fibroblasts cells (V79-4) and BALB/c mice (Kang et al. 2010); hair follicle cells against γ radiation (Kim et al. 2016). Based on the drug likeness (Lipinski rule of fives), phloroglucinol is the only compound that fulfill these criteria. These rules are used to evaluate the likeness of a compound to become a drug, so any compound that follows these rules has a higher chance of becoming a drug.

#### Polysaccharides

Marine extracts sourced from macroalgae mainly contain polysaccharides such as alginate and fucoidan (Fig. 3) that were studied against γ-radiations. There are three macroalgae extract that demonstrated to have radioprotective activities using in vitro and in vivo models with active concentration of 150 µg/ml and 100 mg/kg, respectively. On the other hand, only two macroalgae-derived compounds (sodium alginate and fucoidan) demonstrated to have radioprotective activities using mainly in vivo models. Antioxidative polysaccharide (AP2) fraction from brown algae *Ishige okamurae* was fermented using *Lactobacillus plantarum* with high glucose and mannose content. This fraction increased the survival rate of zebrafish from 47 to 83% when treated with 3.13 µg/ml of AP2 and irradiated with 20 Gy of γ-radiation. When the concentration of AP2 was doubled to 6.25 µg/ml, the ROS and nitric oxide (NO) production decreased from 140 and 160% to about 110 and 120%, respectively (Lee et al. 2017). These results show that AP2 fraction has potential radioprotection and antioxidant activites in reducing the dysfunction of cells in mice. Similarly, fucoidan a sulphated polysaccharide derived from brown algae has also been widely reported with radioprotective activities in in vitro and in vivo studies. Fucoidan has shown radioprotective activities by increasing cell viability and survival rate of bone marrow cells of C57BL/6 and Balb/c mice (Byon et al. 2008); hematopoietic cells of Balb/c mice (Lee et al. 2008); HS68 cells (Lee et al. 2009); and U937 cells and Balb/c mice when exposed to γ-radiation (Rhee and Lee 2011).

Only one radioprotection study was conducted using microalgal extract against γ-radiation. The polysaccharide extract isolated from *Spirulina platensis* demonstrated in vivo activity at 12 mg/kg in dog model (Zhang et al. 2001).

In regards to drug likeness, fucoidan did not follow these rules as it has a large molecular weight, while sodium alginate followed these rules which make it more likely to become a drug candidate.

### UV radiation

UV radiation can be broadly classified into UVA, UVB and UVC and this radiation was substantially reported for cell damage studies in the literature. UVA spectrum ranges from 315 to 400 nm and is considered as the main UV component with ~ 95% of total UV reaches to earth’s surface (Ghissassi et al. 2009; Ridley et al. 2009). Even though UVA is low energetic, it has high penetration capacity and known to be the main environmental cause of skin cancer such as melanoma (Bernerd et al. 2012; Edgar et al. 2011) as it induces DNA damages and mutations via ROS (Fig. 2) (Mouret et al. 2006; Rünger and Kappes 2008; Tyrrell and Keyse 1990). UVB spectrum ranges from 280 to 320 nm (Mackerness 2000) and making only 5% of total UV reaches the earth’s surface due to the ozone layer (Ghissassi et al. 2009; Mackerness 2000). A total of 27 studies (Supplementary Table S1) reported radioprotective activity against UV radiation using marine-derived extracts by either regulating the cell signaling pathway or inhibiting ROS. Interestingly, all these studies were done against UVB radiation apart from one study. Nine of these studies used in vivo models, while the rest of studies used in vitro models. Twenty of these studies were conducted on extracts from macroalgae, six studies on microalgae extracts, and one study on sponge extract, respectively. In terms of pure compounds, 35 studies (Supplementary Table S3) were conducted on compounds derived from marine sources with UV protective activities. Four of these studies were conducted on microalgae, 15 on macroalgae, seven on corals, five on sea cucumbers, and four on sponges.

Ethanol and ethyl extracts from brown (especially *Sargassum* species) and red macroalgae were commonly studied and has shown radioprotective activity against UVB radiation and has reduced apoptosis and ROS concentration according to studies mentioned in Table 1. Ethyl acetate extracts from *Sargassum fulvellum* demonstrated to inhibit the cytotoxicity induced by UVB in HaCaT cells in a concentration ranging from 30 to 100 µg/ml via anti-inflammatory protection against COX-2, TNF-α, and iNOS (Lee et al. 2013a). This study also demonstrated the ability of this extract to provide anti-inflammatory protection in animals using BALB/c mice (Lee et al. 2013a). A study demonstrated that *Polyopes affinis* (Harvey) Kawaguchi and Wang to reduce ROS, cell damage, and apoptosis induced by UVB in HaCaT cells (Hyun et al. 2014). Another study was conducted on *Polysiphonia morrowii* which demonstrated to provide scavenging activity against ROS, apoptosis, and DNA fragmentation induced by UVB in HaCaT cells (Piao et al. 2012).

Extracts from microalgae *Chlorella pyrenoidosa* containing peptides were reported for radioprotective activity against UVC irradiation (15 J/cm^2^) on normal skin fibroblast 966SK (CRL 1881) cells at 1–10 mg/ml. Cells treated at higher extract concentrations showed over 100% cell viability while control gave 70% cell viability as the peptides inhibited the expression of caspase 3, reduced the expression of phosphorylated FADD, cleaved PARP-1 and reduced DNA damage induced by UVC (Shih and Cherng 2012).

In addition, marine microbes alone or isolated from host marine organisms produce pigments that can absorb UV radiation. UV-absorbing bacteria from the mucus of corals were isolated and characterized. The majority of these bacteria belonged to *Firmicutes* (class *Bacilli*) and *Proteobacteria* (class *Gammaproteobacteria*) species which absorbed a wavelength of 208–333 nm at 28 °C and 208–400 nm at 30–34 °C. These results suggest that bacteria hosted by marine animals show UV radiation-absorbing capacity (Ravindran et al. 2013). Studies also suggest that the fluorescent pigments present in corals have potential to dissolve excess radiation energy and filter out damaging UVA and photosynthetically active radiation (PAR) (Salih et al. 2000). Benzylthiocrellidone is a unique sulfur-containing yellow pigment present in *Crella spinulata* (sponge) that has capacity to absorb both UVA (λmax 345 nm) and UVB (λmax 295 nm) (Lam et al. 1999; Pattenden et al. 2002). Pigments produced by iridescent bacterium strain *Cellulophaga fucicola* strain 416 present in Antarctic sponge (Silva et al. 2018), was found to absorb UVB radiation (Silva et al. 2019).

#### Carotenoids

Astaxanthin, 3,3′-dihydroxylated and 4,4′-diketolated (derivative of β-carotene) sourced from microalgae *Haematococcus pluvialis*, *Chlorella vulgaris*, and *Chlorococcum* sp. were reported to stimulate immune system and reduce the free radicals from lipid oxidation through antioxidant activities which helps to protect DNA damage from radiation (Higuera-Ciapara et al. 2006; Xie et al. 2017). Carotenoids from colored marine sponges are also reported to suppress oxygen free radicals and act as photo-protective pigments (Osbourn et al. 2014). In addition, the isolation of radioprotective carotenoids was also reported from lobsters (de Carvalho and Caramujo 2017) and sea cucumbers (Bandaranayake and Rocher 1999).

Seven studies used both in vitro and in vivo models to demonstrate the presence of radioactive properties against UV radiation in carotenoids (Supplementary Table S3). These investigations suggest that carotenes supress ROS and inhibits apoptosis. There are four studies conducted on astaxanthin for its radioprotective activities against UV radiation. One study demonstrated that astaxanthin has potent activity to protect rat kidney fibroblasts from UVA-induced ROS at 10 nmol/L (O'Connorand and O'Brien 1998). Astaxanthin also provides protective activity against UVA-induced DNA damage in 1BR-3 cells, CaCo-2 cells, and HEMAc cells (Lyons and O'Brien 2002), and protects the microorganism against UVB induced photo-oxidation and DNA damage (Sajjad et al. 2017). Astaxanthin also supressed UVC-induced apoptosis and inhibited DNA fragmentation through inhibition of IL-1β and TNF-α expression (Yoshihisa et al. 2014). In this study, astaxanthin (5 µmol/L) alone demonstrated radioprotective activities against UVC (5 mJ/cm^2^) in HaCaT cells by decreasing the level of inducible nitric oxide synthase (iNOS) and cyclooxygenase-2 (COX)-2 (Yoshihisa et al. 2014). On the other hand, fucoxanthin demonstrated to have radioprotective activities in three studies. Fucoxanthin inhibited vascular endothelial growth factor (VEGF) and MMP-13 expression to protect HOS:HR-1 hairless mice from wrinkle formation induced by UVB (Urikura et al. 2011). Fucoxanthin also enhanced the skin barrier protein filaggrin in human dermal fibroblasts and primary skin fibroblastic cells (E15.5 embryo skin) to protect skin against UVA radiation (Matsui et al. 2016); suppressed ROS and inhibited apoptosis in UVB-induced human fibroblast (Heo and Jeon 2009). Due to the large molecular weight of carotenoid compounds, none of these compounds follows Lipinski rule of fives. However, structure modification is a possible way to improve drug properties such as large molecular weight, low solubility, and poor stability (Guo 2017; Yao et al. 2017). As stated before, astaxanthin is not following the rules of drug likeness. Similarly, fucoxanthin is not following these rules to the same reasons (large molecular weight and high LogP value).

#### Mycosporine-like amino acids (MAAs)

In addition to polysaccharides and phlorotannins, marine algae were also studied for the presence of MAAs that were reported for UV-absorbing capacity (Chrapusta et al. 2017). Twenty studies reported in the literature the occurrence of MAA in algae, corals and other marine organisms and that mycosporine–palythine, porphyra and shinorine can absorb UV radiation. The concentration of MAAs differs from the species and the level of UV exposure. In a human study, MMAs were added to cream demonstrating significant improvement in skin firmness and smoothness to human samples (Schmid et al. 2004). The occurrence of MAAs was reported in red algal species that include *Agarophyton chilense*, *Pyropia plicata* and *Champia novae‐zelandiae*. This suggests that seaweeds have developed the capacity to absorb UVA and UVB to withstand harmful rays (Orfanoudaki et al. 2019). The presence and type of MAA in algae vary with climate and other environment conditions (Pangestuti et al. 2018). Studies conducted on *Palmaria palmata*, *Porphyra umbilicalis*, *Porphyra* sp. and *Lichina pygmaea* reported the presence of asterina, mycosporine–serinol, palythine, porphyra and shinorine in these algae and fungi species identified by capillary electrophoresis method (Hartmann et al. 2017). *Pyropia plicata* (red algae) was reported to contain shinorine (~ 3 mg/g DW) and porphyra-334 (4–5 mg/g DW) and the concentration of these stress-induced metabolites varies with environmental changes (Diehl et al. 2019).

A screening of 41 invertebrates from Antarctic marine waters showed the presence of MAAs that include palythine, porphyra-334, shinorine, mycosporine–glycine, palythene, asterina-330, palythinol, and mycosporine–glycine:valine (Karentz et al. 1991). The presence of MAAs was also reported in marine sponge species including *Dysidea herbacea*—produced mycosporine–glutamic acid–glycine (novel MAA) (Bandaranayake et al. 1996); *lsodicfya erinacea*—produced palythinol (McClintock and Karentz 1997); and *Lendenfeldia chondrode—*produced *Lendenfeldia chondrode*-343 and mycosporine–ethanolamine MAAs (Oda et al. 2017). Porphyra-334, shinorine, and mycosporine–glycine were also found in scallop ovaries, which protected human fibroblast cells from UV-induced cell death. In an in vitro study, mycosporine–glycine (EC_50_ = 24 µmol/L), shinorine (EC_50_ = 64 µmol/L), and porphyra-334 (EC_50_ = 294 µmol/L) were reported from strongest to weakest for their UV protection activity (Oyamada et al. 2008). The presence of MAAs differs in colonies (Gleason 1993). The concentration of MAA in marine organisms is linked with the duration and exposure level of UV radiation (Shick et al. 1995, 1999; Ferrier-Pagès et al. 2007) such as the depth (Dunlap and Chalker 1986; Dunlap et al. 1986; Gleason and Wellington 1995; Shick et al. 1995; Corredor et al. 2000), water motion and photosynthetically active radiation (PAR) (Jokiel et al. 1997). Furthermore, the concentration of MAAs secreted by corals varies with coral species and parts (Teai et al. 1998). Even though many compounds from this class follow Lipinski rule of fives such as mycosporine-Gly, mycosporine-2Gly, palythine (Serine/Thr sulfate/Ser-sulfate), palythinol, and asterina-330, the current results have not used cell cultures nor animal models (with the exception of one study). For that reason, more investigations of these compounds in vitro and in vivo are needed to confirm their activities.

#### Phlorotannins

Five studies were conducted on five phlorotannins with UV radioprotective activities (Supplementary Table S3). Three phlorotannins (eckol, dieckol, and phloroglucinol) demonstrated to have radioprotective activities by inhibiting UVB induced photo-oxidative stress in human fibroblasts (Heo et al. 2009). Dieckol was reported to inhibit UVB-induced cytotoxicity in HaCaT cells (Ko et al. 2011). Diphlorethohydroxycarmalol was reported to inhibit UVB in human fibroblast cells via inhibiting ROS and DNA damage (Heo et al. 2010), while fucofuroeckol A protects RBL-2H3 mast cells from UVB via inhibiting histamine release, intracellular calcium, IL-1β and TNF-α and scavenging ROS production (Vo et al. 2018). Phloroglucinol was reported to protect human fibroblast against UVB by inhibiting photo-oxidative stress and DNA damage (Heo et al. 2009); and Balb/c mice skin cells against UVB (Piao et al. 2014). Regarding Lipinski rule of fives, as mention before in γ-radiation, phloroglucinol is the only compound that follow these rules.

#### Polysaccharides

It was reported that polysaccharide fraction (PF) from brown algae *Sargassum muticum* (ethyl acetate extracts) showed radioprotective activity against UVB-induced ROS and apoptosis (Piao et al. 2011). This extract at 12.5–100 µg/ml has shown activity against lipid peroxidation in human keratinocytes (HaCaT cells) (Piao et al. 2011). Later, it was found to inhibit the upregulated metalloproteinase one expression in HaCaT cells induced by UVB in HR-1 mice, and protected cells from wrinkling and photo-aging (Song et al. 2016). PF from *Sargassum fusiforme* has also protected HaCaT cells against UVB by enhancing superoxide dismutase (SOD), GSH-PX and inhibiting ROS, MMP-1, and MMP-9 (Ji et al. 2017); suppressing malondialdehyde (MDA), and activating SOD and catalase (CAT) in hairless Kun Ming (KM) mice (Ye et al. 2018). Furthermore, sulphated-PF from *Hizikia fusiforme* at 50–200 µg/ml reduced UVB-induced ROS in HDF cells, suppressed collagen degradation, and reduced matrix metalloproteinases (MMPs) expression by regulating NF-κB, AP-1, and MAPK signaling (Wang et al. 2018). In regards to macroalgae-derived compounds, carrageenan derived from red algae at 0.78 µg/ml demonstrated to protect normal mouse fibroblast (3T3) cells from UVB-induced DNA damage (Ho et al. 2007). Fucoidan has also demonstrated radioprotective activity against UVB-induced ROS and MDA in HS68 cells (Ku et al. 2010). Due to the large molecular weights of polysaccharide compounds, they did not follow the drug likeness criteria.

### X-ray

An earlier study conducted on the effect of X-ray shows that X-ray workers in China between 1950 and 1995 were at higher risk of cancer due to continuous exposure to IRs (Wang et al. 2002). It was also found that the mortality rate of patients with ankylosing spondylitis increased with single course of X-ray treatment among cancer patients due to the radiation field (Weiss et al. 1994). Only four studies (Tables 2, 3) were conducted on marine-derived extracts/compounds with radioprotective activities against X-ray radiation by down-regulating cell signaling pathway and enhancing immune system. Two of these studies were conducted on one extract and one compound derived from microalgae. Anticancer study conducted on mouse epidermal cells JB6 Cl41 and human malignant melanoma SK-MEL-28 cells against X-ray radiation used laminarin and its sulfate derivatives from *Dictyota dichotoma* (brown algae). They showed X-ray radiation protection by down-regulating MMP-2 and MMP-9 protein kinase activity (Malyarenko et al. 2019). The third study conducted using green algal fraction from *Monostroma angicava* reported to show protective activity against X-ray by immune activation. This fraction (4–16 mg/kg) was used to treat the mice and showed an increase in the count of leukocytes, thrombocytes and erythrocytes of BALB/c mice that were exposed to X-ray irradiation (6 Gy X-rays). This algal fraction also increased the spleen index and natural killer cytostatic activity, hereby suggesting immune activation as the mode of action (Mao et al. 2005). In addition, a polysaccharide extract from the green algae *Ulva pertusa* demonstrated in vivo activity in BALB/c mice by providing antioxidant activity against X-ray (Shi et al. 2013). The last study was conducted on a compound derived from tunicate. An alkaloid extract dendrodoine analog (DA) from *Dendrodoa grossularia* was studied against X-rays (6 MV) using an in vitro-damaged lymphocyte. The results suggested that the antioxidant properties of DA suppressed the toxic effect in treated groups, however, the genetic damage and thiobarbituric acid reactive substances (TBARS) increased in the non-treated group with the increase of radiation dosage (Kalpana et al. 2010). However, until now, the studies conducted against X-rays using marine-based compounds are limited (three studies only) and mainly focus on different signaling pathways in in vitro model. Limited in vitro and in vivo studies call for further investigation on marine-derived compounds to understand the radioprotective mechanism against X-ray radiation. Regarding drug likeness, dendrodoine analog followed these rules while laminarin did not follow these rules.

**Table 2:** Summary of extracts derived from marine organisms showing radioprotective activities against X-ray

Extract	Source	Effective dosage	Radio-protective activity/mechanism	References
Polysaccharide extract	Green algae: *Monostroma angicava* (39)	4–16 mg/kg	Recovered the counts of leukocytes, thrombocytes and erythrocytes of BALB/c mice that were exposed to X-ray irradiation	Mao et al. (2005)
	Green algae: *Ulva pertusa* (40)	4–8 mg/day	Provided antioxidant activity against X-ray in BALB/c mice	Shi et al. (2013)

**Table 3:** Summary of compounds derived from marine organisms showing radioprotective activities against X-ray

Compound	Source	Effective dosage	Radio-protective activity/mechanism	References
Dendrodoine analog (33)	Tunicate: *Dendrodoa grossularia*	6 µg/ml	Provide protection to lymphocytes against damage induced by X-ray radiation via antioxidant activity by reducing lipid peroxidation	Kalpana et al. (2010)
Laminaran and its sulfated derivative (34)	Brown alga: *Dictyota dichotoma*	25–50 µg/ml	Increase cell viability of JB6 Cl41 and SK-MEL-28 cells against X-ray radiation	Malyarenko et al. (2019)

## Compounds with multiple radioprotective activities

In this review, seven compounds have demonstrated to possess multiple radioprotective activities (Table 4). Interestingly, astaxanthin has showed radioprotective activities against γ-ray, UVA, and UVB. Phlorotannin (eckol, dieckol, phloroglucinol, and diphlorethohydroxycarmalol) and fucoidan have demonstrated γ-ray and UVB radioprotection, while fucoxanthin showed radioprotective activities against UVA and UVB. It is worth noting that six of these compounds were derived from macroalgae and one compound from microalgae. This finding might indicate that macroalgae- and microalgae-derived compounds have superior radioprotective activities comparing to other marine sources, and they are more likely to become drugs candidates or nutraceutical supplements to reduce radiation damages.

**Table 4:** Summary of marine-derived compounds that show multiple radioprotective activities

Compound	Activity
Astaxanthin	γ-Ray, UVA, UVB
Eckol	γ-Ray and UVB
Dieckol	γ-Ray and UVB
Phloroglucinol	γ-Ray and UVB
Diphlorethohydroxycarmalol	γ-Ray and UVB
Fucoidan	γ-Ray and UVB
Fucoxanthin	UVA and UVB

Compounds were either natural extracts or commercially purchased

## Conclusion and future prospective

A wide range of marine organisms including macroalgae, microalgae, sponges, sea cucumber and corals have been reported to possess 40 extracts and 34 compounds with potential radioprotective activities against γ-ray, UV and X-ray irradiation. Marine-derived extracts and compounds with radioprotective activity against γ-ray and UV radiation are the most studied radioprotective activities. Algae are the dominant sources of radioprotective compounds reported so far, constituting polysaccharides and pholorotannins. The identified extracts/compunds follow multiple modes of actions that include inhibition of ROS and apoptosis, regulating cell signaling pathways of caspase, MMP, NFK to prevent cell death and increase cell viability. Mycosporine, in particular, MAA-palythine, porphyra and shinorine have unique functional properties to absorb UV radiation and supress the effect of UV damage. However, MAAs presence and activity depend on marine species and environmental factors. Astaxanthin, eckols, phloroglucinol, fucoidan and fucoxanthin were the most studied compounds againt γ and UV radiation. Most of these compounds with high molecular weight could prevent them from becoming a drug candidate, finding active derivatives will be the most logial approach. Though the majority of studies reported in this review were investigated in vitro, however, animals including mice, rat, zebrafish and dog have been used as in vivo models. These animal models confirm that these marine-derived extracts/compounds are effective in protecting against radiation. Only three studies investigated the effect of these marine-derived radioprotective compounds in human subject. These promising results from in vitro and in vivo studies warrant further in-depth research to validate the efficiacy of extracts and compounds in clinical trials as potential radioprotectants. Given ocean accounts for more than 70% of the earth’s surface with a high diversity of marine organisms that are exposed to significant amount of radiation against which they develop resistance. It is with great promise that ocean could become the next frontier for the discovery and development of a new generation marine-derived radioprotectants for human health applications.

## Supplementary Information

Below is the link to the electronic supplementary material.

Supplementary file1 (DOCX 90 KB)
