# Supplementary material for: Advances on marine-derived natural radioprotection compounds: historic development and future perspective
Source: Mar Life Sci Technol. 2021 May 12;3(4):474–87. doi: 10.1007/s42995-021-00095-x (PMC10077276; doi:10.1007/s42995-021-00095-x)
Supplement: Supplementary file 1 — Supplementary file1 (DOCX 90 KB) [file 42995_2021_95_MOESM1_ESM.docx]

**Supplementary Tables**

**Advances on** **marine-derived natural radioprotection compounds: historic development and future perspective**

Reinu E Abraham ^a, 1^*, Mousa Alghazwi ^a, b, 1^, Qi Liang ^a, c^, Wei Zhang ^a^*

^a^ Centre for Marine Bioproducts Development, College of Medicine and Public Health, Flinders University, Bedford Park, SA 5042, Australia

^b^ Department of Pharmaceutical Sciences, College of Clinical Pharmacy, King Faisal University, Al-Ahsa, Kingdom of Saudi Arabia

^c^ Shanxi University of Traditional Chinese Medicine, Taiyuan 030619, China

* Corresponding authors: [wei.zhang@flinders.edu.au](mailto:wei.zhang@flinders.edu.au); [reinu.abraham@flinders.edu.au](mailto:reinu.abraham@flinders.edu.au)

^1^ Equal contributions, joint-first authors

**Supplementary Table S1** Summary of extracts derived from marine organisms showing radio-protective activities against UV radiations

| **Extract** | **Source** | **Effective dosage** | **Radio-protective activity/mechanism** | **Ref.** |
| --- | --- | --- | --- | --- |
| Polysaccharide extracts | Brown algae:  *Hizikia fusiforme*  (Sulfated polysaccharides fraction) (13) | 50 - 200 µg/ml | -Reduced ROS and provide protection against UVB in HDF cells.  -Provided protection to collagen synthesis, and reduced MMPs expression via regulating NF-κB, AP-1, and MAPK signalling | (Wang et al. 2018) |
|  | Brown algae:  *Sargassum fusiforme* (14) | 0.5 - 2 mg/ml | Provided cytoprotection to HaCaT cells against UVB via enhancing SOD and GSH-PX and inhibiting ROS, MMP-1, and MMP-9 | (Ji et al. 2017) |
|  | Brown algae:  *Sargassum fusiforme* | 200 – 600 mg kg^-1^day^-1^ | Protected hairless Kun Ming (KM) mice against UVB by inhibiting ROS and MDA, and activating SOD and CAT | (Ye et al. 2018) |
| Ethanol extracts | Brown algae:  *Sargassum Thallus* (15) | 10 - 100 µg/ml | Protected HS68 cells from cytotoxicity and oxidative stress induced by UVB via activating Type Ⅰ collagen and Type Ⅰ procollagen | (Lee et al. 2012) |
|  | Brown algae:  *Undaria crenata* (16) | 50 – 200 µg/ml | Protected HaCaT cells against UVB induced cell damage and apoptosis via inhibiting oxidative stress | (Hyun et al. 2013) |
|  | Green algae:  *Codium thalli* (17) | 10 – 100 µg/ml | Protected HS68 cells from cytotoxicity and oxidative stress induced by UVB via activating Type Ⅰ collagen and Type Ⅰ procollagen | (Lee et al. 2012) |
|  | Red algae: *Bonnemaisonia hamifera* (18) | 100 µg/ml | Reduced cytotoxicity induced by UVB in HaCaT cells via inhibiting ROS, DNA fragmentation and apoptosis | (Piao et al.,2012a) |
|  | Red algae:  *Lomentaria hakodatensis* Yendo (19) | 100 mg/ml | Protected HaCaT from cellular damage and apoptosis induced by UVB via scavenging activity against ROS | (Kim et al.,2012) |
|  | Red algae:  *Polyopes affinis* (Harvey) Kawaguchi & Wang (20) | 100 µg/ml | Reduced ROS production, cell injury, and apoptosis induced by UVB | (Hyun et al. 2014) |
|  | Red algae:  *Chondracanthus tenellus* (Harvey) Hommersand (21) | 100 µg/ml | Reduced ROS production, injury degree, apoptosis and DNA fragmentation induced by UVB | (Piao et al. 2012b) |
|  | Red algae:  *Polysiphonia morrowii* Harvey (22) | 25 – 100 µg/ml | Protected HaCaT cells against UVB induced apoptosis and DNA fragmentation via reducing ROS | (Piao et al. 2012c) |
| Ethyl acetate extracts | Brown algae: *Sargassum muticum* (23) | 12.5 – 100 µg/ml | Protected HaCaT cells from UVB induced ROS and apoptosis | (Piao et al. 2011) |
|  | Brown algae:  *Sargassum fulvellum* (24) | 30 – 100 µg/ml  3 and 10 µg | Provided anti-inflammatory protection to HaCaT Cells and BALB/c Mice from UVB via inhibiting cytotoxicity, COX-2, TNF-α, and iNOS | (Lee et al. 2013b) |
|  | Brown algae: *Sargassum muticum* (25) | 100 mg/kg | -Protected cells from wrinkling and photoaging induced by UV-B in HR-1 mice  -Inhibited the upregulated metalloproteinase 1 expression induced by UV-B in HaCaT cells | (Song et al. 2016) |
|  | Microalga:  *Ettlia sp.* (26) | YC001  20 µg/ml | Protected normal human dermal fibroblasts cells from UV-B induced cytotoxicity and DNA damage | (Lee et al. 2017a) |
|  | Microalga:  *Spirogyra sp.* (27) | 50-100 µg/ml | Improved cell viability of HaCaT cells after exposing to UV-B and reduced the number of cells in sub-G1 phase  -Reduced ROS formation in zebrafish model | (Wang 2017) |
| Phenolic extracts | Brown algae:  *Macrocystis pyrifera* (28) | 0.04 mg/ml PGE (phloroglucinol equivalents) | Protected zebrafish embryo model from UVB induced dead embryos | (Guinea et al. 2012) |
|  | Red algae:  *Porphyra columbina* (29) | 0.04 mg/ml PGE | Protected zebrafish embryos model from UVB induced dead embryos | (Guinea et al. 2012) |
| Methanol extracts | Red algae:  *Porphyra yezoensis* (30) | 0.5 – 3 mg/ml | Provided cell protection to HaCaT cells from UVB induced cytotoxicity and apoptosis via activating signalling pathways such as JNK and ERK | (Kim et al. 2014c) |
|  | Sponge:  *Dysidea herbace* (31) |  | Had absorption in the UV-B and UV-A region with the spectrum maximum at 328 nm | (Bandaranayake et al. 1996) |
| Water extracts | Red algae:  *Euchema cottoni* (32) | The content of the seaweed in the cream was 0.5% | Adding seaweed extract, cocoa polyphenol, and *aloe vera* as active ingredient in cream to male Wistar rats demonstrated to decrease wrinkle and erythema levels induced by UVB | (Ristanti et al. 2018) |
| *Chlorella* derived peptides | Microalgae:  *Chlorella* (33) | 5-10 mg/ml | Protected normal skin fibroblast 966SK (BCRC 60153) from UVB via inhibiting the expression of CYR61 and MCP-1 | (Chen et al. 2011) |
|  | Microalgae:  *Chlorella pyrenoidosa* (34) | 1-10 mg/ml | Protected normal skin fibroblast 966SK (CRL 1881)) from UVC via inhibiting the expression of caspase 3 and Phosphorylated FADD | (Shih and Cherng 2012) |
| Hexane extracts | Microalgae:  *Chlorella vulgaris* (35) | 20 µg/ml | Protected cells from UV-B and promoted DNA repair properties in normal human dermal fibroblasts | (Kim et al. 2014b) |
| Other extracts | Microalga:  *Arthrospira platensis* (36) | 20 µg/ml | -Protected normal human dermal fibroblasts cells from UV-B induced cytotoxicity, DNA damage and reverse G2/M phase cell cycle arrest  -Inhibited the upregulated MMP1 and MMP3 expression induced by UV-B in nHDF cells | (Lee et al. 2017a) |
|  | Brown algae (37)  (A mixture of polyphenols/ 2-O-(2,4,6-trihydroxyphenyl)-6,6́-bieckol (16.5%), dieckol (15.7%), 7-phloroeckol (8.1%), 8,8́-bieckol (6.2%), 6,6́-bieckol (6.1%), phlorofurofukoeckol (3.7%), eckol (3.5%), 2-phloroeckol (2.4%)) | 3 – 6 mg | Protected SKH-1 mice from UVB by decreasing tumor multiplicity, tumor volume and COX-2 | (Hwang et al. 2006) |
|  | Red algae:  *Gelidium amansii* (38)  (Fermented *Gelidium amansii* and *Cirsium japonicum* extract mixture) | 10 µg/ml  500 mg /kg body weight | -Activated Type I procollagen levels and reduced matrix MMP-1 expression induced by UVB in Hs68 cells  -Reduced wrinkles and inhibited the expression of MMP-13, MMP-2 and MMP-9 enhanced by UVB in SKH-1 hairless mice | (Kim et al. 2014a) |

**Supplementary Table S2** Summary of compounds derived from marine organisms showing radio-protective activities against γ radiations

| **Compound** | **Source** | **Effective dosage** | **Radio-protective activity/mechanism** | **Ref.** |
| --- | --- | --- | --- | --- |
| Astaxanthin (1) | Commercial: Sigma | 20 µg/ml | Reduced mutagenic effect of γ radiation on human peripheral blood lymphocytes | (Pilinska et al. 2016) |
|  | Commercial: Sigma | 20 µg/ml | Provided cytogenetic protection to human peripheral blood lymphocytes (from worker who exposed to high radiation in Chornobyl Nuclear Power) that exposed to γ radiation (1 Gy) as it reduced chromosomal abnormalities from 25.7% to 15.3% and from 32.6% to 18.4% in two group patients | (Kurinnyi et al. 2016) |
|  | Commercial: Aladdin Co. (Shanghai, China) | 50 mg/kg | -Protected hematopoietic system against total body irradiation (TBI) with 8 Gy γ-ray that induce bone marrow suppression in C57BL/6 mice  -Reduced apoptosis and ROS generation induced by TBI | (Xue et al. 2017) |
|  | Sigma, United States | 20 µg/ml | -Protected human blood lymphocytes from γ radiation  -Decreased DNA damage and apoptosis level | (Kurinnyia et al. 2017) |
| β-carotene (2) | Microalgae:  *Dunaliella bardawil* | 40 mg | Provided radio-protective activity to children exposed to Chernobyl accident (γ radiation) by reducing lipid peroxidation | (Ben-Amotz et al. 1998) |
| Eckol (3) | Brown algae:  *Ecklonia cava* | 10 mg/kg | -Decreased the mortality of C57BL/6 mice induced by ionizing radiation through enhancing hematopoietic recovery and repairing damaged DNA  -Protected C57BL/6 mice from γ radiation (7 Gy) induced apoptosis through inhibiting P53 and Bax, and increasing Bcl-2 expression | (Park et al. 2008) |
|  | Brown algae:  *Ecklonia cava* | 10 µg/ml | Protected Chinese hamster lung fibroblasts (V79-4) from γ radiation (10 Gy) induced cytotoxicity by inhibiting ROS and apoptosis via inhibiting Bax, caspase 3, and caspase 9 and activating cytochrome C and Bcl-2 | (Zhang et al. 2008) |
| Eckol and phloroglucinol | Brown algae:  *Ecklonia cava* | 20 mg/kg | Improved survival rate of ICR mice exposed to γ radiation (8 Gy) via inhibiting ROS | (Moon et al. 2008) |
| Dieckol (4) | Brown algae:  *Ecklonia cava* | 10-25 mg/kg | -Increased survival rate in C57BL/6 mice after exposing to γ radiation (7 – 9 Gy)  -Restored bone marrow progenitor cells and inhibited DNA damage and lipid peroxidation after exposing to γ radiation | (Park et al. 2010) |
| Diphlorethohydroxycarmalol (5) | Brown algae:  *Ishige okamurae* | 10 µg/ml  100 mg/kg | Protected Chinese hamster lung fibroblast (V79-4) cells from γ radiation (10 Gy) via inhibiting apoptosis and ROS | (Ahn et al. 2011) |
| Phloroglucinol (6) | Commercial:  Brown algae:  *Ecklonia cava* | 10 µg/ml  50 mg/kg | Protected Chinese hamster lung fibroblasts (V79-4) cells and BALB/c mice against ROS induced by γ radiation through inhibiting apoptosis via inhibiting caspase and JNK | (Kang et al. 2010) |
|  | Brown algae:  *Ecklonia cava* | 25-100 µg/ml | Protected splenocytes of C57BL/6 mice from γ radiation (2 Gy) induced cytotoxicity via inhibiting ROS, apoptosis, and DNA damage | (Park et al. 2011) |
|  | Brown algae:  *Ecklonia cava* | 10 mg/kg | Protected small intestinal crypt cells in C57BL/6 mice from γ radiation (7Gy) by inhibiting apoptosis via inhibiting P53, Bax and Bak and activating Bcl-2 and Bcl-X_S/L_ | (Ha et al. 2013) |
|  | Brown algae:  *Ecklonia cava* | 10 mg/1.5 cm^2^ | Protected hair follicles cells from apoptosis and DNA damage induced by γ radiation in C57BL/6 mice via inhibiting P53 and cleaved | (Kim et al. 2016) |
| Triphlorethol-A (7) | Brown algae:  *Ecklonia cava* | 30 µmol/L | Reduced intracellular H_2_O_2_ induced by γ radiation by inhibiting apoptosis and DNA damage via reducing the expression of Bax and caspase 3 and increase Bcl-2 expression in V79- 4 cells | (Wu Won et al. 1995) |
| Sodium Alginate (8) |  |  | Protected esophageal mucosa of ICR mice against γ radiation | (Hasegawa et al. 1989) |
| Fucoidan (9) | Commercial: Sigma  Brown algae:  *F. vesiculosus* | 50 µg/ml | Increased cell viability of bone marrow cells of C57BL/6 and Balb/c mice exposed to γ radiation via inhibiting apoptosis | (Byon et al. 2008) |
|  | Commercial: Sigma  Chemical:  Brown algae: *F. vesiculosus* | 100 mg/kg | Increased survival rate and hematopoietic cells induced by γ radiation in Balb/c mice | (Lee et al. 2008) |
|  | Commercial:  Heawon Biotech | 10–100 µg/ml | Increased the survival rate of HS68 cells after exposing to γ radiation | (Lee et al. 2009) |
|  | Commercial:  Heawon Biotech | 1–100 µg/ml  1-100 mg/kg | -Protected U937 cells from cytotoxicity induced by γ radiation  -Increased survival rate and different haematological parameters induced by γ radiation in Balb/c mice | (Rhee and Lee 2011) |

**Supplementary Table S3** Summary of compounds derived from marine organisms showing radio-protective activities against UV radiations

| **Compound** | **Source** | **Effective dosage** | **Radio-protective activity/mechanism** | **Ref.** |
| --- | --- | --- | --- | --- |
| Astaxanthin | Commercial: F. Hoffmann-La Roche | 10 nmol/L | Protected rat kidney fibroblasts from ROS induced by UVA via enhancing CAT and SOD | (O'Connor and O'Brien 1998) |
|  | Commercial: Sigma | 10 µmol/L | Protected 1BR-3 cells, CaCo-2 cells, and HEMAc cells from DNA damage induced by UV-A via inhibiting SOD and activating GSH | (Lyons and O'Brien 2002) |
|  | Commercial: Fuji Chemical Industry Co. Ltd (Toyama, Japan) | 5 µmol/L | -Reduced iNOS and cyclooxygenase-2 induced by UVB and UVC in HaCaT cells  -Reduced apoptosis level through inhibiting IL-1β and TNF-α expression | (Yoshihisa et al. 2014) |
|  | Bacterium: *Deinococcus* sp. strain WMA-LM9 | 41.6 µg/ml | Protected the microorganism against UV-B induced photo-oxidation and DNA damage | (Sajjad et al. 2017) |
| Fucoxanthin (10) | Brown algae:  *Sargassum siliquastrum* | 5-250 µmol/L | Protected human fibroblast from UV-B induced cytotoxicity through decreasing the production of ROS and inhibiting apoptosis | (Heo and Jeon 2009) |
|  | Brown algae:  *Undaria pinnatifida* | 0.001% fucoxanthin solution | Protected HOS:HR-1 hairless mice from wrinkle formation induced by UV-B through inhibiting VEGF and MMP-13 expression | (Urikura et al. 2011) |
|  | Brown algae:  *Undaria pinnatifida* | 0.1-10 µmol/L | Protected skin from UV-A radiation through enhancing skin barrier protein filaggrin in human dermal fibroblasts and primary skin fibroblastic cells (E15.5 embryo skin) | (Matsui et al. 2016) |
| Eckol, dieckol, and phloroglucinol | Brown algae:  *Ecklonia cava* | 5-100 µmol/L | Inhibited photo-oxidative stress induced by UV-B in human fibroblast. Dieckol was found to work via inhibiting DNA damage | (Heo et al. 2009) |
| Dieckol | Brown algae:  *Ecklonia cava*  (Ethyl acetate fraction) | 100 µg/ml  50 µmol/L | -Protected HaCaT cells against UVB induced cytotoxicity and apoptosis  - Reduced ROS and NO in zebrafish model | (Ko et al. 2011) |
| Diphlorethohydroxycarmalol | Brown algae:  *Ishige okamurae* | 5-250 µmol/L | Reduced ROS induced by UV-B via inhibiting DNA damage in human fibroblast cells | (Heo et al. 2010) |
| Fucofuroeckol-A (11) | Brown algae:  *Ecklonia stolonifera*  Okamura | 12.5 – 50 µmol/L | Provided protection to RBL-2H3 mast cells against UVB by inhibiting histamine release, intracellular calcium, IL-1β and TNF-α and scavenging ROS production | (Vo et al. 2018) |
| Phloroglucinol | Commercial: Sigma | 10-50 mg/kg | Protected Balb/c mice skin cells from ROS and apoptosis induced by UVB via inhibiting Bax and caspase 3 | (Piao et al. 2014) |
| Carrageenan (12) | Red algae | 0.78 µg/ml  (k-carrageenan) | Protected normal mouse fibroblast (3T3) cells from the DNA damage induced by UVB | (Ho et al. 2007) |
|  | Commercial:  *Eucheuma spinosum*, commercial, and *Eucheuma cottonii*, respectively | 6.25 – 100 µg/ml | Protected HaCaT cells from UVB induced cytotoxicity and ROS | (Thevanayagam et al. 2014) |
| Fucoidan | Brown algae | 10 – 100 µg/ml | Protected HS68 cells from ROS and MDA induced by UVB | (Ku et al. 2010) |
| Butanol and ethyl acetate fractions, clerosterol (compound) (13) | Green algae:  *Codium fragile* | 30 – 100 µg/ml  1 – 3 µg/ml | -Protected HaCaT cells from UVB induced cytotoxicity and inhibited the level of COX-2, iNOS, NO, and TNF-α  -Provided protection to BALB/c mice by inhibiting COX-2, iNOS, TNF-α, and lipid peroxidation | (Lee et al. 2013a) |
| Fucosterol (14) | Brown algae:  *Hizikia fusiformis* | 0.1 – 10 µg/ml | Decreased MMP, IL-6, and production of Type I procollagen expression induced by UVB via modulating AP-1 and TGF-β1 signalling | (Hwang et al. 2014) |
| Protein extract (calmodulin) (15) | Green algae:  *Ulva rigida* | 50 µg/ml | Protected cortical astrocytes from UVB by increasing cell viability and reducing lipid peroxidation | (Zehlila et al. 2017) |
| Mycosporine-like amino acid (MAA) Porphyra-334 (16) | Red algae:  *Porphyra umbilicalis* | 0.1-5 µg/ml  5% MAA in a cream | Protected mouse fibroblasts from UVA  -Provided protection to human against UVA as it inhibits lipid peroxidation and skin ageing | (Schmid et al. 2004) |
|  | Coral:  *Fungia repanda*;  *Pocillopora eydouxi*;  *Pocillopora meandrina*;  *Stylophora pistillata* |  | Absorb λ_max_ 334nm | (Dunlap et al. 1986;Corredor et al. 2000) |
|  | Sea cucumber Epidermal tissues:  *Holothuria atra* |  |  | (Bandaranayake and Rocher 1999) |
| Mycosporine-Gly (17)  Mycosporine–2Gly (18)  Palythine (Serine/Thr sulfate/Ser-sulfate) (19)  Palythinol (20) | Sponge:  *Dysidea herbace* |  | Absorb λ_max_ 310 nm (Mycosporine-Gly) | (Bandaranayake et al. 1996) |
|  | Coral:  *Acropora Formosa;*  *Acropora microphthalma;*  *Acropora danaï;*  *Agaricia agaricites;*  *Acropora cervicornis;*  *Fungia repanda;*  *Fungia scutaria;*  *Fungia repanda;*  *Lobophyllia hemprichii;*  *Lobophyllia hemprichii;*  *Mycetophyllia ferox;*  *Pocillopora eydouxi*;  *Pocillopora meandrina*;  *Porites astreoides*;  *Pocillopora damicornis:*  *Stylophora pistillata*; |  | Absorbs λ_max_ 310 nm (Mycosporine-Gly)  Absorb λ_max_ 331nm (Mycosporine–2Gly)  Absorb λ_max_ 320 nm and 321nm (Palythine (Serine/Thr sulfate/Ser-sulfate))  Absorb λ_max_ 332nm (Palythinol)  UV-light absorption spectra for the above MAAs overlap from 285 to 350 nm in coral *Acropora spp*. | Dunlap and Chalker, 1986; (Dunlap et al. 1986;Gleason 1993;Shick et al. 1995;Wu Won et al. 1995;McClintock and Karentz 1997;Shick et al. 1999; Lam et al. 1999;Corredor et al. 2000;Pattenden et al. 2002) Maoka and Akimoto 2008) |
|  | Sea cucumber:  *Holothuria atra* |  |  | (Bandaranayake and Rocher 1999) |
| Mycosporine-Glutamic acid-Glycine (21) | Sponge:  *Dysidea herbace* |  | Absorb λ_max_ 330 nm | (Bandaranayake et al. 1996) |
| Mycosporine-NMA (serine/ threonine) (22 -23) | Coral:  *Stylophora pistillata* |  | Absorb λ_max_ 325nm and λ_max_ 328nm | (Dunlap et al. 1986) |
| Mycosporine (methylamine:serine/ threonion) (24) | Coral:  *Pocillopora eydouxi*  *Pocillopora meandrina*; *Pocillopora damicornis; Stylophora pistillata* |  | Absorb λ_max_ 330nm | (Corredor et al. 2000;Reef et al. 2009) |
| Asterina-330 (25) | Coral:  *Acropora microphthalma*;  *Porites astreoides*;  *Porites astreoides* |  |  | (Dunlap and Chalker 1986;Gleason 1993;Pattenden et al. 2002) |
|  | Sea cucumber:  *Holothuria atra* |  |  | (Bandaranayake and Rocher 1999) |
| Shinorine (26) | Coral:  *Acropora cervicornis;*  *Fungia repanda;*  *Lobophyllia hemprichii;*  *Mycetophyllia ferox;*  *Porites astreoides*;  *Pocillopora eydouxi;*  *Pocillopora meandrina;*  *Stylophora pistillata*; |  | Absorb λ_max_ 334nm | (Gleason 1993;Teai et al. 1998;Shick et al. 1999;Corredor et al. 2000) |
|  | Sea cucumber:  *Holothuria atra* |  |  | (Bandaranayake and Rocher 1999) |
| Benzylthiocrellidone (27) | Sponge:  *Crella spinulata* |  | Absorption of both UV-A (λ_max_ 345 nm) and UV-B (λ_max_ 295 nm) regions | (Bandaranayake et al. 1996;Llewellyn and Airs 2010) |
| Usujirene (28) | Sponge:  *Dysidea herbace* |  | Absorbs λ_max_ 354 nm | (Bandaranayake et al. 1996) |
| 6-deoxygadusol (29)  Homarine (30)  Trigonelline (31) | Sea cucumber Ovaries/ Viscera:  *Holothuria atra* |  |  | (Bandaranayake and Rocher 1999) |
| Palythine-threonine (MAA) (32) | Coral:  *Pocillopora capitata* |  | Absorbs λ_max_ 320 nm | (Carignan et al. 2009) |

**References**

Ahn M, Moon C, Yang W, Ko EJ, Hyun JW, Joo HG, Jee Y, Lee NH, Park JW, Ko RK, Kim GO, Shin, T (2011) Diphlorethohydroxycarmalol, isolated from the brown algae *Ishige okamurae*, protects against radiation-induced cell damage in mice. Food Chem Toxicol 49:864-870

Carignan MO, Cardozo KHM, Oliveira-Silva D, Colepicolo P, Carreto JI (2009) Palythine–threonine, a major novel mycosporine-like amino acid (MAA) isolated from the hermatypic coral *Pocillopora capitata*. J Photoch Photobio B 94:191-200

Chen CL, Liou SF, Chen SJ, Shih MF (2011) Protective effects of *Chlorella*-derived peptide on UVB-induced production of MMP-1 and degradation of procollagen genes in human skin fibroblasts. Regul Toxicol Pharmacol 60:112-119

Guinea M, Franco V, Araujo-Bazán L, Rodríguez-Martín I, González S (2012) *In vivo* UVB-photoprotective activity of extracts from commercial marine macroalgae. Food Chem Toxicol 50:1109-1117

Hasegawa T, Takahashi T, Inada Y, Yamada C, Tanaka Y (1989) Reparative effects of sodium alginate (Alloid G) on radiation stomatitis. Nippon acta radiologica (Nihon Igaku Hoshasen Gakkai zasshi) 49:1047-1051

Hwang E, Park SY, Sun ZW, Shin HS, Lee DG, Yi TH (2014) The protective effects of fucosterol against skin damage in UVB-irradiated human dermal fibroblasts. Mar Biotechnol 16:361-370

Hwang H, Chen T, Nines RG, Shin HC, Stoner GD (2006) Photochemoprevention of UVB-induced skin carcinogenesis in SKH-1 mice by brown algae polyphenols. Int J Cancer 119:2742-2749

Hyun YJ, Piao MJ, Kim KC, Zheng J, Yao CW, Cha JW, Kang HK, Yoo ES, Koh YS, Lee NH, Ko MH, Hyun JW (2014) Photoprotective effect of a Polyopes affinis (Harvey) Kawaguchi and Wang (Halymeniaceae)-derived ethanol extract on human keratinocytes. Trop J Pharm Res 13:863-871

Kim AD, Piao MJ, Hyun YJ, Kang HK, Suh IS, Lee NH, Hyun JW (2012) Photo-protective properties of Lomentaria hakodatensis yendo against ultraviolet B radiation-induced keratinocyte damage. Biotechnol Bioprocess Eng 17:1223-1231

Kim HM, Lee DE, Park SD, Kim YT, Kim YJ, Jeong JW, Lee JH, Jang SS, Chung DK, Sim JH, Huh CS (2014a) Preventive effect of fermented Gelidium amansii and Cirsium japonicum extract mixture against UVB-induced skin photoaging in hairless mice. Food Sci Biotechnol 23:623-631

Kim K, Lee J, Heo J, Cho D, Kim H, Kim K (2014b) The extract of Chlorella vulgaris exerts protective effects against ultraviolet B radiation-induced damages in human dermal fibroblasts. Kor J Aesthet Cosmetol 12:479-486

Kim S, You DH, Han T, Choi EM (2014c) Modulation of viability and apoptosis of UVB-exposed human keratinocyte HaCaT cells by aqueous methanol extract of laver (Porphyra yezoensis). J Photochem Photobiol B 141:301-307

Lee C, Jang JH, Kim B, Park CI (2012) Anti-aging effects of marine natural extracts against UVB-induced damages in human skin
cells. J Soc Cosmet Sci Korea 38:255–261
Lee C, Park GH, Ahn EM, Kim BA, Park CI, Jang JH (2013a) Protective effect of Codium fragile against UVB-induced pro-inlammatory and oxidative damages in HaCaT cells and BALB/c mice. Fitoterapia 86:54–63
Lee JJ, Kim K, Heo J, Cho DH, Kim HS, Han S, Ahn K, An IS, An S, Bae S (2017a) Protective efect of Arthrospira platensis extracts
against ultraviolet B-induced cellular senescence through inhibition of DNA damage and matrix metalloproteinase-1 expression
in human dermal ibroblasts. J Photochem Photobiol B 173:196–203

Maoka T, Akimoto N (2008) Carotenoids and their fatty acid esters of spiny lobster *Panulirus japonicus*. J Oleo Sci 57:145-152

Park E, Ahn G, Yun JS, Kim MJ, Bing SJ, Kim DS, Lee J, Lee NH, Park JW, Jee Y (2010) Dieckol rescues mice from lethal irradiation by accelerating hemopoiesis and curtailing immunosuppression. Int J Radiat Biol 86:848-859

Piao M, Hyun Y, Cho S, Kang H, Yoo E, Koh Y, Lee N, Ko M, Hyun J (2012a) An ethanol extract derived from *Bonnemaisonia hamifera* scavenges ultraviolet B (UVB) radiation-induced reactive oxygen species and attenuates UVB-induced cell damage in human keratinocytes. Mar Drugs 10:2826-2845

Piao MJ, Hyun YJ, Oh TH, Kang HK, Yoo ES, Koh YS, Lee NH, Suh IS, Hyun JW (2012b) *Chondracanthus tenellus* (Harvey) hommersand extract protects the human keratinocyte cell line by blocking free radicals and UVB radiation-induced cell damage. In Vitro Cell Dev Biol.-Animal 48:666-674

Reef R, Kaniewska P, Hoegh-Guldberg O (2009) Coral skeletons defend against ultraviolet radiation. PLoS ONE 4:e7995

Ristanti EY, Ramlah S, Indriana DD (2018) Antiaging properties of cream made with cocoa polyphenol, Aloe vera (*Aloe barbadensis*) and seaweed (*Euchema cottoni*) as active agents. Jurnal Industri Hasil Perkebunan 13:43-52

Thevanayagam H, Mohamed SM, Chu WL (2014) Assessment of UVB-photoprotective and antioxidative activities of carrageenan in keratinocytes. J Appl Psychol 26:1813-1821

Wang L, Ryu B, Kim WS, Kim Gh, Jeon YJ (2017) Protective effect of gallic acid derivatives from the freshwater green alga *Spirogyra* sp. against ultraviolet B-induced apoptosis through reactive oxygen species clearance in human keratinocytes and zebrafish. Algae 32:379-388

Wu JJW, Rideout JA, Chalker BE (1995) Isolation and structure of a novel mycosporine-like amino acid from the reef-building corals *Pocillopora damicornis* and *Stylophora pistillata*. Tetrahedron Lett 36:5255-5256

Zehlila A, Schaumann A, Mlouka AB, Bourguiba I, Hardouin J, Masmoudi O, Cosette P, Amri M, Jouenne T (2017) Glioprotective effect of *Ulva rigida* extract against UVB cellular damages. Algal Res 23:203-215
